# LncRNA LGALS8-AS1 Promotes Breast Cancer Metastasis Through miR-125b-5p/SOX12 Feedback Regulatory Network

**DOI:** 10.3389/fonc.2021.711684

**Published:** 2021-10-22

**Authors:** Duanyang Zhai, Tianfu Li, Runyi Ye, Jiong Bi, Xiaying Kuang, Yawei Shi, Nan Shao, Ying Lin

**Affiliations:** ^1^ Breast Disease Center, The First Affiliated Hospital of Sun Yat-sen University, Guangzhou, China; ^2^ Laboratory of Surgery, The First Affiliated Hospital, Sun Yat-Sen University, Guangzhou, China

**Keywords:** LGALS8-AS1, metastasis, EMT, SOX12, feedback, miR-125b-5p

## Abstract

**Background:**

Metastasis is a major factor weakening the long-term survival of breast cancer patients. Increasing evidence revealed that long non-coding RNAs (lncRNAs) were involved in the occurrence and development of breast cancer. In this study, we aimed to investigate the role of LGALS8-AS1 in the metastatic progression of breast cancer cells and its potential mechanisms.

**Results:**

The lncRNA LGALS8-AS1 was highly expressed in breast cancer and associated with poor survival. LGALS8-AS1 functioned as an oncogenic lncRNA that promoted the metastasis of breast cancer both *in vitro* and *in vivo*. It upregulated SOX12 *via* competing as a competing endogenous RNA (ceRNA) for sponging miR-125b-5p and acted on the PI3K/AKT signaling pathway to promote the metastasis of breast cancer. Furthermore, SOX12, in turn, activated LGALS8-AS1 expression *via* direct recognition of its sequence binding enrichment motif on the LGALS8-AS1 promoter, thereby forming a positive feedback regulatory loop.

**Conclusion:**

This study manifested a novel mechanism of LGALS8-AS1 facilitating the metastasis of breast cancer. The LGALS8-AS1/miR-125b-5p/SOX12 reciprocal regulatory loop dyscrasia promoted the migration and invasion of breast cancer cells. This signaling axis could be applicable to the design of novel therapeutic strategies against this malignancy.

## Introduction

Breast cancer, derived from the epithelial tissues of the breast gland, is the most common diagnosed cancer and the primary cause of cancer-related death in women around the world (1). Although many advances have been made in the diagnosis and treatment of breast cancer in recent decades, metastasis remains the main challenge that reduces the survival rate and the quality of life of patients (2). Breast cancer metastasis is responsible for most breast cancer mortality. It is reported that breast cancer may spread to distant organs *via* metastasis, and 90% of breast cancer mortalities are attributable to metastasis (3). Although treatment for breast cancer has been studied for many years, the prognosis of metastatic breast cancer remains poor. The molecular mechanisms of the metastasis of breast cancer remain unclear. Therefore, it is necessary to thoroughly explore the key molecular mechanisms of breast cancer and speed up improving effective strategies against this disease.

Metastasis is the overwhelming cause of death in breast cancer patients. It is known that epithelial–mesenchymal transition (EMT) is intently related to the metastasis of breast cancer. Cancer cells can undergo the EMT process, which may facilitate metastasis. Tumor metastasis is a complex multistep process, and EMT is an early and important step of metastasis (4). Epithelial cells decrease their polarity and cell–cell adhesion during the EMT process and change into mesenchymal cells (5). This allows cells to gain migratory and invasive properties (6–8). In tumors, EMT is associated with increased aggressiveness and poor prognosis, emphasizing its mechanistic role in tumor progression and metastasis (9, 10). Due to the change of cell characteristics, some marker proteins may change concomitantly during the EMT process. Decreased E-cadherin and increased N-cadherin/vimentin/SNAIL1 expressions are conventional EMT markers (11). This suggests a strong association between the EMT program and breast cancer progression due to the frequent expressions of the EMT markers in invasive breast cancer (12).

Long non-coding RNA (lncRNA), more than 200 nucleotides (nt) in length, is an emerging subgroup of ncRNAs that functions at the transcriptional and post-transcriptional levels. It has a critical role in cell invasion, migration, metastasis, proliferation, and apoptosis in breast cancer. The occurrence of human malignancies frequently accompanies lncRNA dysregulation (13). In recent years, accumulating studies have revealed that non-coding RNAs play a key role in tumor development and metastasis (14). For example, the overexpression of lncRNA p10247 is correlated with metastasis in breast cancer (15). A high expression of the lncRNA HOTAIR in breast cancer tumors is a critical predictor of metastasis (16). At present, there is no related research on LGALS8-AS1, and its role in breast cancer is not clear. However, it remains unknown with regard to various novel lncRNAs in the biological regulation of breast cancer metastasis.

LncRNAs commonly regulate their downstream target genes by acting as competing endogenous RNAs (ceRNAs) to sponge microRNA (miRNA), thereby affecting the proliferation and metastasis of various cancer types (17–19). For instance, linc-ZNF469-3 enhanced the invasion ability of triple-negative breast cancer through the miR-574-5p–ZEB1 signaling axis (20). A low expression of nuclear factor kappa B (NF-κB) interacting lncRNA (NKILA) is associated with breast cancer metastasis (21). NKILA binds to NF-kB/IkB, leading to the inhibition of IκB kinase (IKK)-induced IκB phosphorylation and NF-κB activation, and suppresses cancer metastasis. LncRNA associated with breast cancer brain metastasis (BCBM) (Lnc-BM) could activate the JAK2/STAT3 signaling pathway and promote BCBM (22). The study of Zhang et al. suggested that the miR-125b-5p/STAT3 pathway could serve as a potential target for the treatment of cancers associated with dysregulated mTORC1 (23). Besides, miR-125b targets the matrix metalloproteinase MMP13 to inhibit the proliferation, migration, and invasion of cutaneous squamous cell carcinoma (24). Through bioinformatics analysis, we found that miR-125b-5p might form ceRNA with LGALS8-AS1.

SOX12 is a member of the highly conserved SRY-related high-motility group box transcription factor family (25). Recent data demonstrated that SOX12 was related to prognostic survival in breast cancer (26). It was reported that SOX12 was upregulated and contributed to the initiation and progression of several cancers, such as gastric cancer (27), colorectal cancer (28), hepatocellular carcinoma (29), and lung cancer (30). However, there are only a few studies on the effect of SOX12 on breast cancer metastasis.

In this study, we identified a breast cancer metastasis-related lncRNA, LGALS8-AS1. LGALS8-AS1 was highly expressed in breast cancer and predicted poor prognosis. *In vitro* and *in vivo* experiments indicated that LGALS8-AS1 promoted breast cancer cell metastasis. It also acted as the ceRNA, sponging miR-125b-3p to enhance the expression of SOX12. In addition, LGALS8-AS1 could activate the PI3K/AKT pathway and was feedback loop transcriptionally regulated by SOX12 in breast cancer. Our study elucidated the molecular regulatory mechanism of LGALS8-AS1 in breast cancer and provided a prognostic indicator as well as a capable target for the treatment of breast cancer patients.

## Materials and Methods

### Human Breast Clinical Specimens and Cell Lines

Breast cancer and adjacent tissue specimens were obtained from patients who underwent surgery at The First Affiliated Hospital of Sun Yat-sen University. All patients provided full consent for participation in the study. The study was approved by the Committees for Ethical Review of The First Affiliated Hospital of Sun Yat-sen University. The human breast epithelial cell line (MCF10A) and the breast cancer cell lines (MDA-MB-231, MCF7, AU565, SK-BR-3, and BT474) were obtained from ATCC (Rockville, MD, USA) and cultured in Dulbecco’s modified Eagle’s medium (DMEM) (Gibco, Carlsbad, CA, USA) containing 10% fetal bovine serum (Gibco). All cells were maintained in an atmosphere with 5% CO_2_ and at 37°C. The pcDNA3.1-LGALS8-AS1 and pcDNA3.1-SOX12 overexpression plasmids were purchased from RiboBio (Guangzhou, China). pGPH1-sh-LGALS8-AS1 and pGPH1-sh-SOX12 were purchased from GenePharma, Co., Ltd. (Shanghai, China). The mimics and inhibitor of hsa-miR-125b-5p were purchased from RiboBio. Cell transfection was performed using Lipo3000 reagent according to the manufacturer’s instructions.

### RNA Isolation and Real-Time Quantitative PCR

LncRNAs and messenger RNAs (mRNAs) were extracted from the cells using TRIzol^®^ (Invitrogen, Thermo Fisher Scientific, Inc., Waltham, MA, USA) and miRNAs were extracted with a miRNA Extraction Kit obtained from Tiangen (Beijing, China) according to the manufacturer’s instructions. A PrimeScript™ RT Reagent kit (TaKaRa Bio, Inc., Shiga, Japan) was used to synthesize complementary DNA (cDNA) from the lncRNA and mRNA. miRNAs were reverse transcribed using a miRNA reverse transcription kit (TaKaRa Bio Inc.). Real-time quantitative PCR (RT-qPCR) analysis was performed in triplicate for each sample using TB Green^®^ Fast qPCR Mix (TaKaRa Bio Inc.) with GAPDH/U6 as the endogenous control. A two-step cycling condition was selected for RT-qPCR. The following thermocycling conditions were used: 1 cycle at 95°C for 30 s, followed by 40 cycles of 95°C of denaturation for 5 s, 60°C combined annealing and extension stages for 10 s, and maintenance at 4°C. Data were analyzed using the 2^−ΔΔCq^ method. The primers used are shown in **Supplementary Table S1**.

### Western Blot Assay

Total protein extracted from breast cancer cells and tissues were lysed using RIPA lysis buffer (Beyotime, Shanghai, China) containing phosphatases and protease inhibitor. The BCA Protein Assay Kit (Beyotime) was used to detect the concentration of protein according to the manufacturer’s instructions. Then, the lysates were separated by 10% SDS gel electrophoresis and transferred into a polyvinylidene fluoride (PVDF) membrane (Millipore, Burlington, MA, USA). The membrane was blocked with 5% bovine serum albumin (BSA) at room temperature for 2 h, followed by incubation with primary antibodies at 4°C overnight. The primary antibodies used were as follows: anti-SOX12 (dilution, 1:1,000; ab54371, Abcam, Cambridge, UK), anti-GAPDH (1:10,000; cat. no. 60004-1-Ig, Proteintech Group, Inc., Wuhan, China), anti-AKT (1:1,000; 4691S, Cell Signaling Technology, Danvers, MA, USA), anti-p-AKT (1:1,000; 4060S, Cell Signaling Technology), anti-N-cadherin (1:500; 13116S, Cell Signaling Technology), anti-E-cadherin (1:500; 14472S, Cell Signaling Technology), anti-vimentin (1:500; 5741T, Cell Signaling Technology), anti-SNAIL1 (1:500; 3879S, Cell Signaling Technology), and anti-p-P85 (1:1,000; ab138364, Abcam). Subsequently, the membrane was incubated with the secondary antibodies (BOSTER Biological, Wuhan, China) for 1.5 h at room temperature. Protein bands were detected using electrochemiluminescence (ECL) assay reagents (Beyotime).

### Subcellular Fractionation and Fluorescence *In Situ* Hybridization

Nuclear and cytoplasmic lncRNA was separated using the NE-PER™ Nuclear and Cytoplasmic Extraction Reagents (Invitrogen) and was subjected to quantitation using RT-qPCR assay. For fluorescence *in situ* hybridization (FISH) assays, the cells were immobilized, permeabilized, and hybridized with 20 μM Cy3-labeled LGALS8-AS1 probe mix (RiboBio). The cell nuclei were stained with DAPI (Sigma-Aldrich, St. Louis, MO, USA). Intracellular distribution of Cy3-labeled LGALS8-AS1 was observed by fluorescence microscopy (Olympus IX81, Tokyo, Japan).

### RNA Immunoprecipitation Assay

RNA immunoprecipitation (RIP) assay was carried out using the Magna RIP RNA-Binding Protein Immunoprecipitation Kit following the manufacturer‘s protocol (Millipore). In brief, the cells were lysed in a RIP lysis buffer, and then the supernatant was transferred to a nuclease-free tube on ice and the resuspended beads were added, which were incubated with Ago2 or IgG. The bead-bound immunoprecipitate was eluted with an elution buffer and the purified RNA fraction analyzed by RT-qPCR.

### Luciferase Reporter Assay

Sequences of the wild-type or mutant LGALS8-AS1 fragment or SOX12 3′-UTR containing the predicted binding sites of miR-125b-5p were sub-cloned into a psiCHECK2 dual-luciferase vector (Promega Corporation, Madison, WI, USA). The luciferase reporter plasmids were co-transfected into MDA-MB-231 and MCF-7 cells with miR-125b-5p mimics or negative control (NC) using Lipofectamine^®^ 3000 (Invitrogen, Thermo Fisher Scientific, Inc.) following the manufacturer’s instructions, and the transfected cells were cultured at 37°C in a humidified incubator with 5% CO_2_ for 36 h. Luciferase signals were measured using the Dual-Glo^®^ Luciferase Assay System (Promega Corporation) according to the manufacturer’s instructions. The activity of luciferase was detected with the Synergy 2 Multidetector Microplate Reader (BioTek Instruments Inc., Winooski, VT, USA).

### Migration and Invasion Assays

The migration and invasion assays were performed using 24-well Transwell plates. Appropriate amounts of cells with serum-free conditioned medium were seeded into the upper chamber, either uncoated or coated with Matrigel (BD Biosciences, San Jose, CA, USA). Complete medium was added into the lower chambers. After maintaining for 24 h at 37°C, the cells on the upper chamber were removed and the invaded cells were treated with 4% paraformaldehyde (FD, Hangzhou, China) and stained with crystal violet. The cells were then observed and counted under a microscope.

### Wound Healing Assay

The MDA-MB-231 and MCF-7 cells were seeded into six-well plates and cultured to the sub-confluent state. After starvation in serum-free DMEM (Gibco, Thermo Fisher Scientific, Inc.) for 24 h, a straight wound was scratched at the bottom of the plate with a 200-μl sterile pipette tip. After gently rinsing, the cells were cultured in serum-free medium for 24 h. Cell migration was observed and calculated at 0 and 24 h, using an inverted light microscope. Scratch healing (%) = (initial scratch area − final scratch area)/initial scratch area × 100.

### Assessment of Breast Cancer Metastasis *In Vivo*

The breast cancer metastasis assay was conducted in female nude mice (5–6 weeks old). All the procedures involving the animal experiments were performed in accordance with the Guide for the Administration of Affairs Concerning Experimental Animals. Cells (1 × 10^6^) were injected into the tail vein of nude mice. The lungs were removed 4 weeks after inoculation and metastatic nodules were counted. The whole lung was removed and fixed in paraformaldehyde, embedded in paraffin, and subjected to hematoxylin–eosin staining. The numbers of lung metastatic foci were calculated to evaluate the development of pulmonary metastasis.

### Statistical Analyses

Data in this research were presented as the mean ± standard deviation. Comparisons between two groups were conducted using unpaired Student’s *t*-tests. One-way ANOVA followed by Tukey’s *post-hoc* test was used for comparisons to one control group. Statistical analysis was performed using SPSS (IBM SPSS 23.0, IBM Corp., Armonk, NY, USA). Survival data were estimated using the Kaplan–Meier method and analyzed using the log-rank test. Spearman’s correlation analysis was used to calculate the correlations in the expressions of LGALS8-AS1/miR-12b-5p/SOX12. All experiments were repeated three times. *P* < 0.05 was considered to indicate a statistically significant difference.

## Results

### LGALS8-AS1 Is Specifically Upregulated in Breast Cancer Tissues and Cell Lines

**Figure 1:**
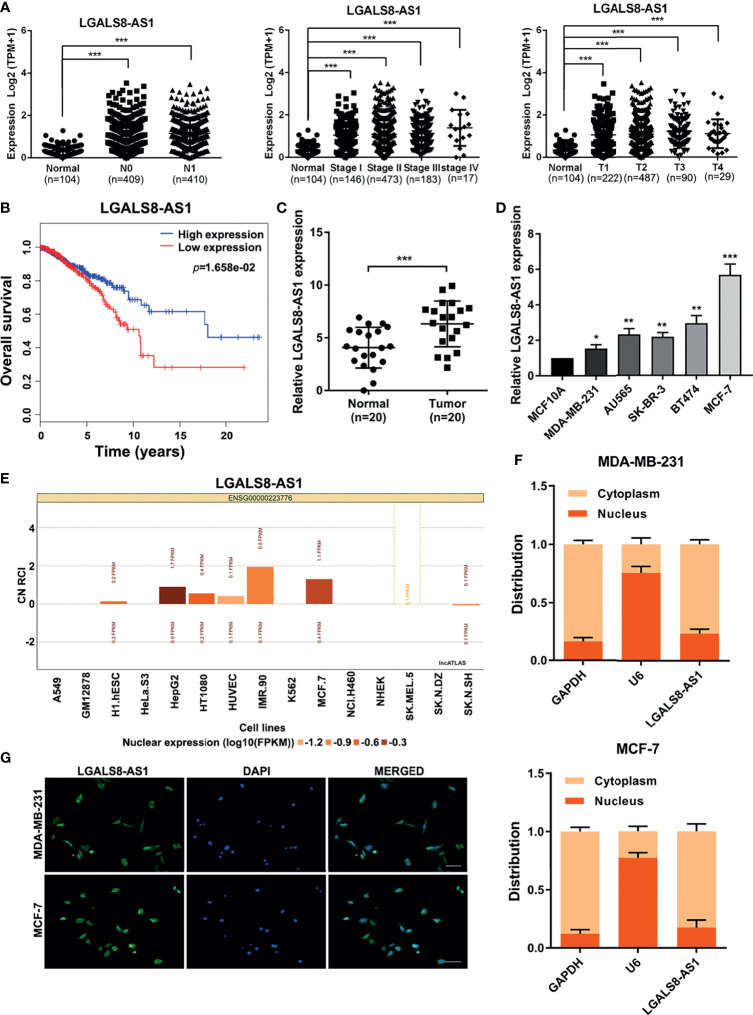
The lncRNA LGALS8-AS1 is specifically upregulated in breast cancer tissues and cell lines. **(A)** Analysis of LGALS8-AS1 expression among different clinical stages performed in the breast cancer gene (BRCA) dataset of The Cancer Genome Atlas (TCGA). **(B)** Kaplan–Meier plotter for LGALS8-AS1 expression in TCGA BRCA dataset. **(C)** Expression of LGALS8-AS1 detected in breast cancer tissues and corresponding paracancerous tissues using real-time quantitative PCR (RT-qPCR). **(D)** Expression of LGALS8-AS1 detected in different breast cancer cell lines using RT-qPCR. **(E)** Subcellular localization of LGALS8-AS1 in different cells queried in the lncATLAS database. **(F)** LGALS8-AS1 expression in the nucleus and cytoplasm of MDA-MB-231 and MCF-7 cells detected by RT-qPCR. **(G)** RNA fluorescence *in situ* hybridization (RNA-FISH) assay performed to detect the subcellular localization of LGALS8-AS1 in breast cancer cells. LGALS8-AS1 is stained in *green* and nuclei are stained in *blue* (DAPI). *p < 0.05, **p < 0.01, ***p < 0.001.

In order to identify the role of LGALS8-AS1 in breast cancer, we analyzed the RNA sequencing (RNA-seq) profiles of breast cancer tissues and normal tissues from The Cancer Genome Atlas (TCGA) dataset. As shown in **Figure 1A**, LGALS8-AS1 expression in breast tissues was higher than that in normal tissues (N0/N1 *vs.* normal, stages I/II/III/IV *vs.* normal, and T1/T2/T3/T4 *vs.* normal). Moreover, Kaplan–Meier analysis illustrated a remarkably poorer survival in the group with higher LGALS8-AS1 expression (**Figure 1B**). Additionally, we collected the matched fresh breast cancer tissues and normal adjacent tissues in surgery. RT-qPCR was performed to verify the expression level of LGALS8-AS1 in 20 pairs of breast cancer tissues and adjacent normal tissues. The results demonstrated that LGALS8-AS1 was upregulated in breast cancer tissues (**Figure 1C**). Subsequently, we confirmed that the expression of LGALS8-AS1 in various breast cancer cell lines (MDA-MB-231, AU565, SK-BR-3, BT474, and MCF-7) was similarly higher than that in mammary epithelial cell (MCF10A) by RT-qPCR (**Figure 1D**).

As the function of lncRNA is relative to its subcellular distribution, we predicted the subcellular localization of LGALS8-AS1 using the lncATLAS database (http://lncatlas.crg.eu/). LGALS8-AS1 was mainly distributed in the cytoplasm of the available cells (**Figure 1E**). Furthermore, RT-qPCR analysis of LGALS8-AS1 expression in the nucleus and cytoplasm showed that it was mainly expressed in the cytoplasm of both MDA-MB-231 and MCF-7 cells, which was confirmed by the FISH assay (**Figures 1F, G** **)**. These results indicated that LGALS8-AS1 was significantly increased in breast cancer and mainly located in the cytoplasm of breast cancer cells.

### LGALS8-AS1 Promotes the Metastasis of Breast Cancer Cells *via* EMT

**Figure 2:**
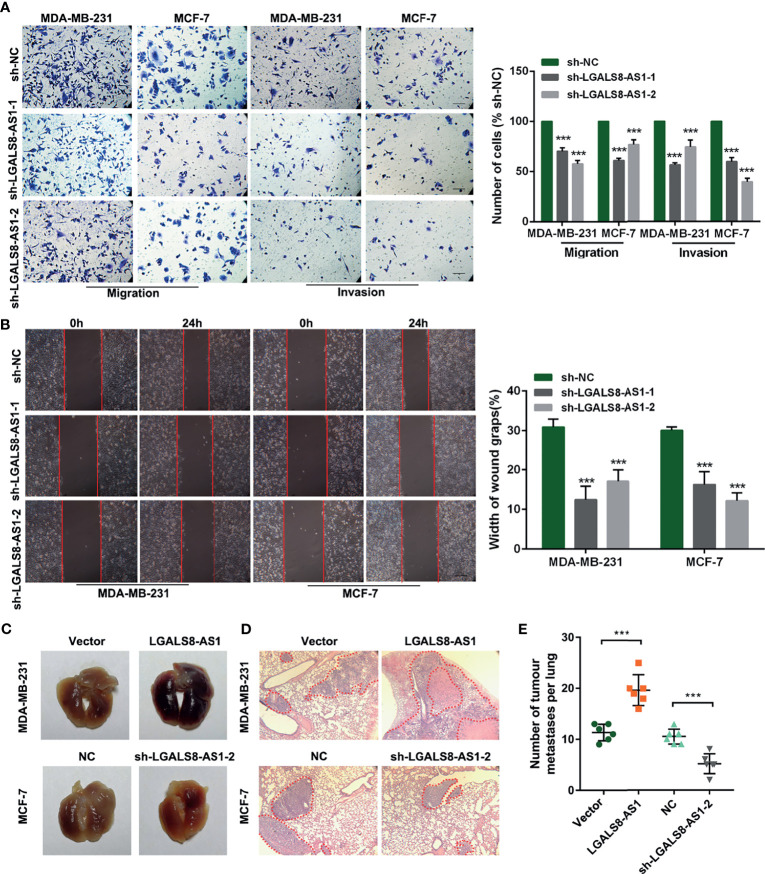
LGALS8-AS1 promotes the invasion and metastasis of breast cancer cells. **(A, B)** Invasive and metastatic capacities detected in MDA-MB-231 and MCF-7 cells with LGALS8-AS1 knockdown using Transwell and scratch wound assays. **(C, D)** Representative images of macroscopic metastatic nodules in the lung and hematoxylin–eosin (HE) staining results of a mouse lung metastasis model constructed with MDA-MB-231 and MCF-7 cells with LGALS8-AS1 stable overexpression or knockdown. **(E)** Number of tumor metastatic nodules counted in the mouse lung metastasis model. ***p < 0.001.

To further evaluate the effect of LGALS8-AS1 on the metastatic ability of breast cancer cells, we transfected MDA-MB-231 and MCF-7 cells with two diverse short hairpin RNAs (shRNAs) against LGALS8-AS1 (sh-LGALS8-AS1-1 and sh-LGALS8-AS1-2) or an LGALS8-AS1-overexpressing plasmid (pcDNA-LGALS8-AS1). The transfection efficiency was confirmed by RT-qPCR analysis (**Supplementary Figure S1A**). Transwell migration, invasion, and wound healing assays revealed that LGALS8-AS1 silencing significantly inhibited the metastasis of breast cancer cells (**Figures 2A, B** **)**. On the contrary, the opposite influence was observed with LGALS8-AS1 overexpression in MDA-MB-231 and MCF-7 cells (**Supplementary Figures S1B, C**). Besides, the lung metastasis model showed that the number of metastatic nodules formed in the lungs was extraordinarily increased in the LGALS8-AS overexpression group and reduced in the LGALS8-AS knockdown group (**Figures 2C–E**).

**Figure 3:**
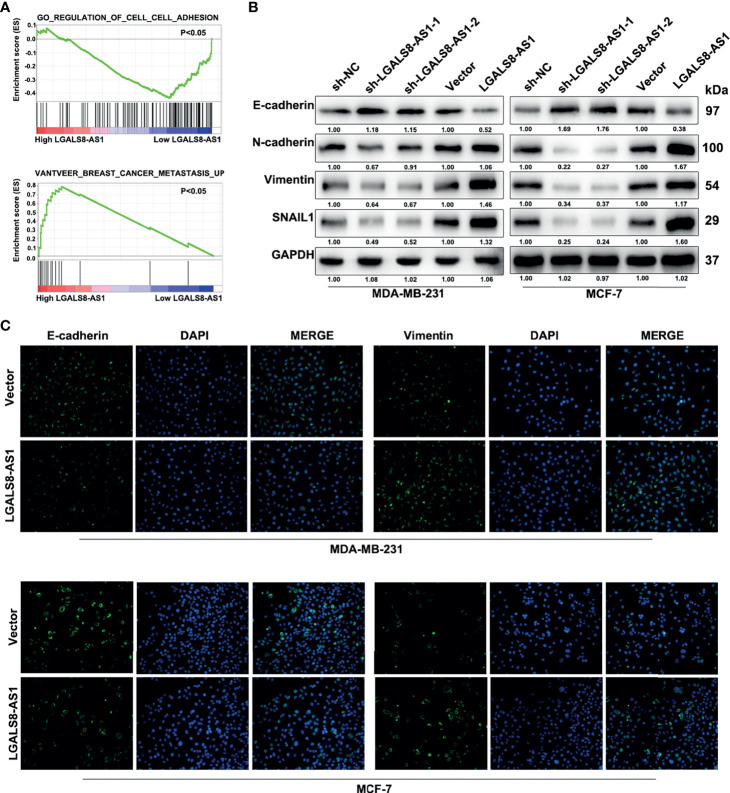
LGALS8-AS1 promotes the metastasis of breast cancer cells *via* epithelial–mesenchymal transition (EMT). **(A)** Gene set enrichment analysis (GSEA) revealed that LGALS8-AS1 was related to cell adhesion and metastasis in breast cancer. **(B, C)** Effect of LGALS8-AS1 knockdown or overexpression on the expressions of EMT-related proteins detected in MDA-MB-231 and MCF-7 cells using Western blot and immunofluorescence assays.

Furthermore, gene set enrichment analysis (GSEA) based on the breast cancer dataset from TCGA revealed that a high LGALS8-AS1 expression was associated with cell junction and breast cancer metastasis (**Figure 3A**). Western blot and immunofluorescence staining showed that LGALS8-AS1 overexpression significantly decreased the expression of E-cadherin, whereas it increased the levels of N-cadherin, vimentin, and SNAIL in both MDA-MB-231 and MCF-7 cells. On the contrary, silencing of LGALS8-AS1 increased the expression level of E-cadherin and suppressed the expressions of N-cadherin, vimentin, and SNAIL (**Figures 3B, C** **)**. Taken together, these results indicated that LGALS8-AS1 functioned as a metastasis facilitator in breast cancer cells *via* the EMT process.

### LGALS8-AS1 Binds With miR-125b-5p to Act as a ceRNA in Breast Cancer Cells

**Figure 4:**
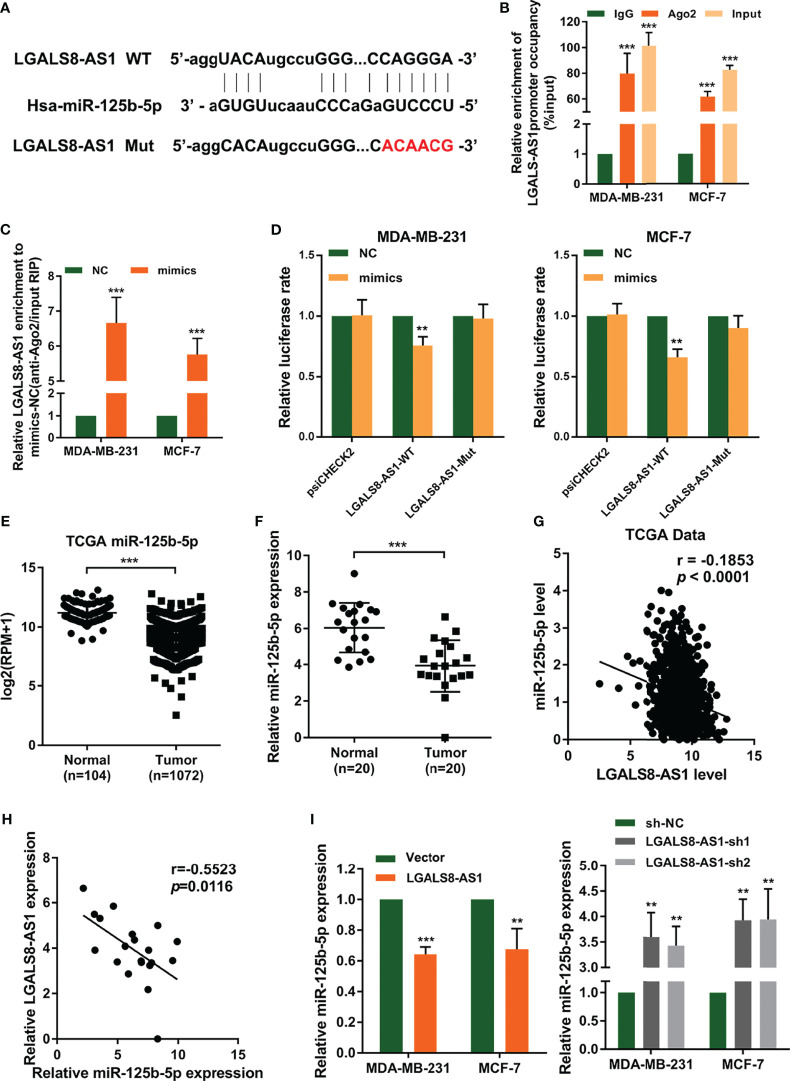
LGALS8-AS1 acts as a sponge of miR-125b-5p in breast cancer. **(A)** Schematic diagram of the miR-125b-5p putative binding sites and mutant site in LGALS8-AS1 predicted by the LncBase database. **(B, C)** Relative enrichment of LGALS8-AS1 and miR-125b-5p associated with Ago2 in MCF-7 and MDA-MB-231 and MCF-7 cells detected using anti-AGO2 RIP (nonspecific IgG as the negative control). **(D)** Luciferase activities in MDA-MB-231 and MCF-7 cells after co-transfection of the LGALS8-AS1 mutant or wild-type (WT) plasmid together with the miR-125b-5p mimics or miR-NC. **(E)** Expression of miR-125b-5p analyzed between tumor and normal tissues using the breast cancer gene (BRCA) dataset of The Cancer Genome Atlas (TCGA). **(F)** The expression of miR-125b-5p was detected in breast cancer tissues and corresponding paracancerous tissues using real-time quantitative PCR (RT-qPCR). **(G)** Correlation between the expressions of LGALS8-AS1 and miR-125b-5p in TCGA BRCA dataset. **(H)** Correlation between the expressions of LGALS8-AS1 and miR-125b-5p in clinical breast cancer tissues. **(I)** Effect of LGALS8-AS1 knockdown or overexpression on the expression of miR-125b-5p detected in MDA-MB-231 and MCF-7 cells by RT-qPCR. **p < 0.01, ***p < 0.001.

In order to investigate the regulation mechanism of LGALS8-AS1 in breast cancer, we conducted bioinformatics analysis to predict the assumed downstream targets of LGALS8-AS1. It was found that miR-125b-5p, which contains the complementary binding sites in the LGALS8-AS1 seed region (**Figure 4A**), might be the candidate miRNA to bind to LGALS8-AS1. Also, the Ago-RIP assay demonstrated that the endogenous LGALS8-AS1 was enriched in Ago2-RIPs compared to the control IgG-RIPs in both MDA-MB-231 and MCF-7 cells (**Figure 4B**), and the enrichment of LGALS8-AS1 was significantly higher in the miR-125b-5p overexpression group than that in the control group (**Figure 4C**). Dual-luciferase reporter assays showed that the overexpression of miR-125b-5p decreased the luciferase activity of LGALS8-AS wild-type transfection, but not the LGALS8-AS-Mut transfection in MDA-MB-231 and MCF-7 cells (**Figure 4D**). The expression level of MiR-125b-5p was downregulated in breast cancer tissues compared with that in the normal tissues, confirmed by the breast cancer gene (BRCA) dataset of TCGA and fresh frozen clinical samples (**Figures 4E, F** **)**. In addition, a negative correlation between the expression levels of LGALS8-AS1 and miR-125b-5p in breast cancer tissues was verified by Pearson’s correlation analysis (**Figures 4G, H** **)**. Moreover, the RT-qPCR assays demonstrated that miR-125b-5p expression was markedly elevated in breast cancer cells transfected with LGALS-AS1-sh1 or LGALS-AS1-sh2 compared to the control group. Inversely, miR-125b-5p expression was downregulated in LGALS-AS1 overexpressed cells (**Figure 4I**). These results indicated that LGALS-AS1 directly “sponged” miR-125b-5p in breast cancer.

### MiR-125b-5p Directly Suppresses SOX12 in Breast Cancer Cells

**Figure 5:**
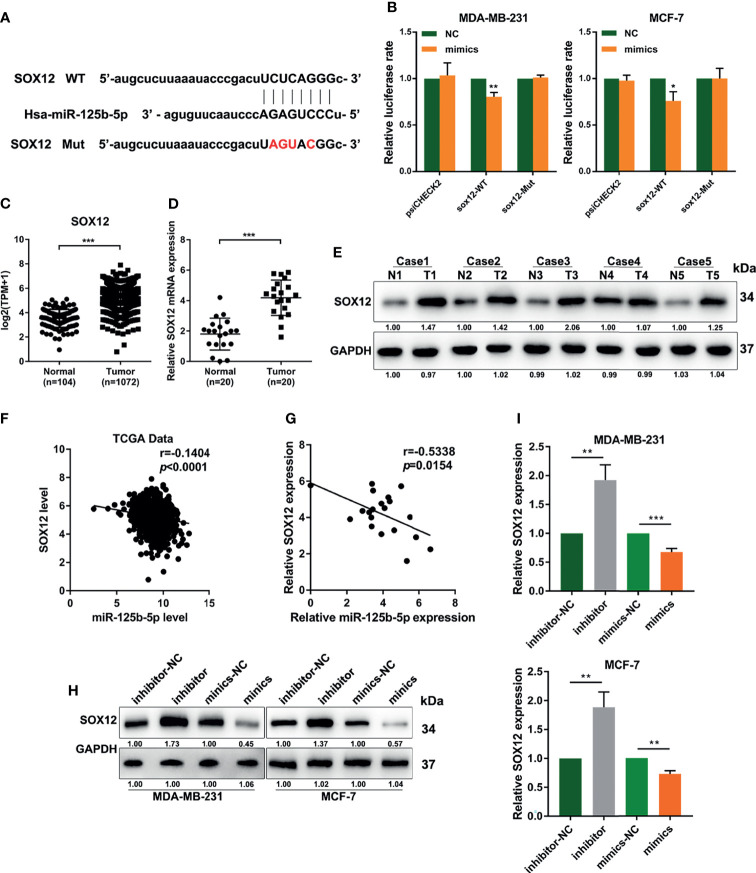
miR-125b-5p directly binds to the 3′-UTR of CRABP2 and suppresses SOX12 expression in breast cancer cells. **(A)** Schematic diagram of the miR-125b-5p putative binding sites and mutant site in the 3′-UTR of SOX12 predicted by bioinformatics. **(B)** Luciferase activities in MDA-MB-231 and MCF-7 cells after co-transfection of the SOX12 mutant or wild-type (WT) plasmid together with the miR-125b-5p mimics or miR-NC. **(C)** The expression of SOX12 was analyzed between tumor and normal tissues using the breast cancer gene (BRCA) dataset of The Cancer Genome Atlas (TCGA). **(D, E)** mRNA and protein expressions of SOX12 detected in breast cancer tissues and corresponding paracancerous tissues using RT-qPCR and Western blot assays. **(F)** Correlations between the expressions of SOX12 and miR-125b-5p in TCGA BRCA dataset. **(G)** Correlations between the expressions of SOX12 and miR-125b-5p in clinical breast cancer samples. **(H, I)** mRNA and protein expressions of SOX12 detected in MDA-MB-231 and MCF-7 cells with the miR-125b-5p mimics or inhibitor transfection. *p < 0.05, **p < 0.01, ***p < 0.001.

To explore the genes regulated by miR-125b-5p in breast cancer, we predicted its target genes using the starBase database (http://starbase.sysu.edu.cn/index.php) (**Figure 5A**). We found that miR-125b-5p could bind to the 3′-UTR region of *SOX12*, which is a key gene that promotes tumor metastasis. Then, we performed dual-luciferase reporter assays and found that miR-125b-5p decreased the luciferase activity with SOX12 wild type (SOX12-WT) transfection, but not the SOX12 mutant (SOX12-Mut) group (**Figure 5B**). We analyzed the dataset from TCGA and concluded that the expression of SOX12 in breast cancer tissues was higher than that in normal tissues (**Figure 5C**), which was further verified by the RT-qPCR analysis and Western blot assay in clinical samples (**Figures 5D, E** **)**. Moreover, the results of Pearson’s correlation analyses indicated a negative correlation between the expression levels of miR-125b-5p and SOX12 in breast cancer (**Figures 5F, G** **)**. In addition, Western blot (**Figure 5H**) and RT-qPCR (**Figure 5I**) assays demonstrated that the expression of SOX12 was markedly upregulated in MDA-MB-231 and MCF-7 cells transfected with the miR-125b-5p inhibitor and downregulated when transfected with the miR-125b-5p mimics. Taken together, miR-125b-5p directly suppressed the expression of SOX12 by binding to the 3′-UTR region in breast cancer cells.

### LGALS8-AS1 Promotes SOX12-Mediated Metastasis by Decoying miR-125b-5p

**Figure 6:**
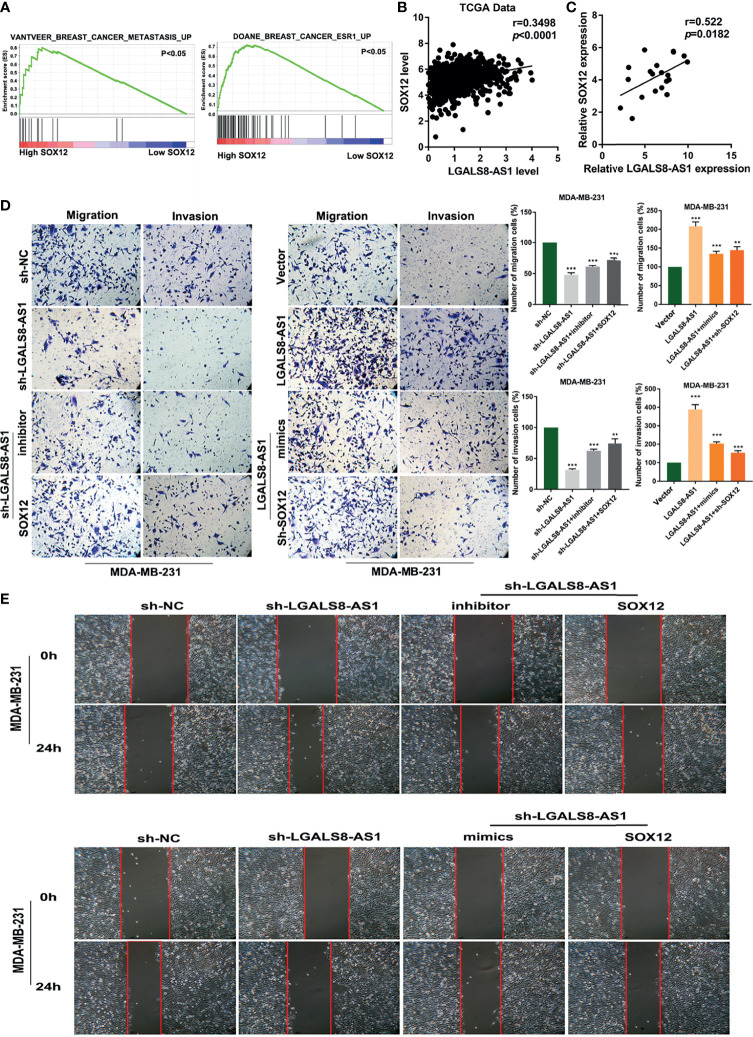
LGALS8-AS1 promoted SOX12-mediated metastasis through decoying hsa-miR-125b-5p. **(A)** Gene set enrichment analysis (GSEA) revealed that SOX12 was related to metastasis in breast cancer. **(B)** Correlation between the expressions of SOX12 and LGALS8-AS1 in the breast cancer gene (BRCA) dataset of The Cancer Genome Atlas (TCGA). **(C)** Correlations between the expressions of SOX12 and LGALS8-AS1 in clinical breast cancer samples. **(D, E)** Combined effect of LGALS8-AS, miR-125b-5p mimics, and sh-SOX12 on the cell migration and invasion of MDA-MB-231 and MCF-7 cells determined by Transwell and scratch assays. **p < 0.01, ***p < 0.001.

GSEA based on the dataset from TCGA (**Figure 6A**) suggested that a high SOX12 expression was related to tumor metastasis in breast cancer. Pearson’s correlation analysis confirmed a positive relationship between the expression levels of LGALS8-AS1 and SOX12 in TCGA database and in breast cancer tissues (**Figures 6B, C** **)**. The transfection efficiency of the SOX12 overexpression plasmid and the sh-SOX12 plasmid was verified by RT-qPCR. The mRNA expression of SOX12 was increased with transfection of the SOX12 overexpression plasmid, while it was decreased with transfection of the sh-SOX12 plasmid in MDA-MD-231 and MCF-7 cells (**Supplementary Figure S2**). RT-qPCR assays demonstrated that the level of SOX12 was elevated by LGALS8-AS1, which could be partially abolished by the miR-125b-5p mimics. Consistently, the miR-125b-5p inhibitor could reverse the downregulated level of SOX12 by silencing LGALS8-AS1 (**Supplementary Figure S3**). Furthermore, the Transwell migration and invasion assays, as well as the wound healing assay, revealed that LGALS8-AS1 overexpression significantly elevated the metastatic ability of MDA-MB-231 and MCF-7 cells, which could be reversed by the miR-125b-5p mimics or sh-SOX12 (**Figures 6D, E** **)**. These results demonstrated that LGALS8-AS1 promoted the metastasis of breast cancer *via* the LGALS8/AS1-miR-125b-5p/SOX12 axis.

### LGALS8-AS1 Activates the PI3K/AKT Signaling Pathway and Is Feedback Transcriptionally Regulated by SOX12

**Figure 7:**
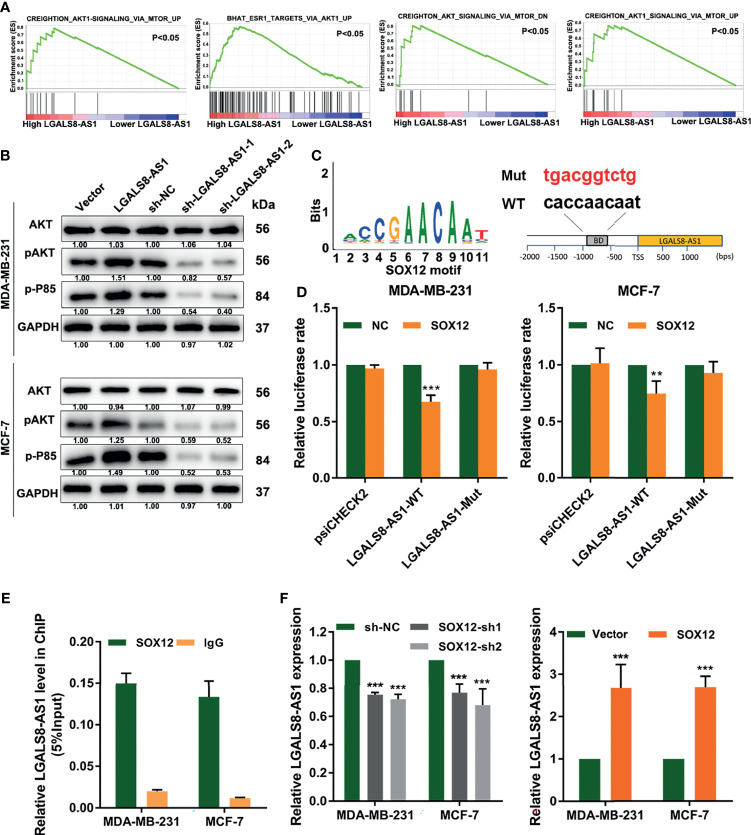
LGALS8-AS1 activated the PI3K/AKT signaling pathway and is feedback transcriptionally regulated by SOX12. **(A)** Gene set enrichment analysis (GSEA) revealed that LGALS8-AS1 expression was related to the PI3K/AKT signaling pathway in breast cancer. **(B)** Effect of LGALS8-AS1 on the expressions of PI3K/AKT signaling-related proteins detected by Western blot assay in MDA-MB-231 and MCF-7 cells. **(C)** The SOX12 motif binding to the promoter of LGALS8-AS1 predicted by JASPAR. **(D)** Luciferase activities in MDA-MB-231 and MCF-7 cells after co-transfection of the SOX12 overexpression plasmid together with the wild-type or mutated LGALS8-AS1. **(E)** Chromatin immunoprecipitation (ChIP) assay demonstrating the direct binding of SOX12 to the LGALS8-AS1 promoter in MDA-MB-231 and MCF-7 cells. **(F)** Effect of SOX12 on the expression of LGALS8-AS1 detected in MDA-MB-231 and MCF-7 cells by real-time quantitative PCR (RT-qPCR). **p < 0.01, ***p < 0.001.

To further determine the mechanism underlying LGALS8-AS1 in breast cancer, we performed GSEA based on the dataset from TCGA and found that LGALS8-AS1 expression was related to the PI3K/AKT signaling pathway (**Figure 7A**). Western blot assays manifested that LGALS8-AS1 could activate the PI3K/AKT signaling pathway. The expressions of p-AKT and p-P85 were elevated with LGALS8-AS1 overexpression transfection, while they were decreased with sh-LGALS8-AS1 transfection (**Figure 7B**). Furthermore, database prediction showed that the SOX12 motif could bind to the promoter of LGALS8-AS1 (**Figure 7C**). We then performed a luciferase reporter gene assay to determine whether LGALS8-AS1 was a direct target of SOX12. As shown in **Figure 7D**, SOX12 significantly decreased the luciferase activity of the LGALS8-AS1 reporter gene in the WT group, whereas the activity of the mutant group was not affected. Furthermore, the chromatin immunoprecipitation quantitative real-time PCR (ChIP-qPCR) assay confirmed that SOX12 promoted the mRNA transcription of LGALS8-AS1 in MDA-MB-231 and MCF-7 cells (**Figure 7E**). The RT-qPCR assays demonstrated that pcDNA-SOX12 markedly elevated the expression of LGALS8-AS1 compared to that in the empty vector, and sh-SOX12 plasmid transfection could inhibit the level of LGALS8-AS1 in MDA-MB-231 and MCF-7 cells (**Figure 7F**). These results demonstrated that LGALS8-AS1 activated the PI3K/AKT signaling pathway and was feedback transcriptionally regulated by SOX12.

## Discussion

Accumulating evidence suggests that lncRNA dysregulation affects the tumorigenesis and metastasis in a variety of cancers. LncRNAs are crucial for cancer proliferation, migration, and invasion *via* regulating tumor-associated genes (31). Growing evidence illustrates that lncRNAs can regulate mRNA expression by competitively binding to the shared miRNAs that block target mRNAs (32, 33). Therefore, lncRNAs are promising biomarkers for diagnosis and potential therapeutic targets. Hu et al. found that lncRNA-HGBC was upregulated in gallbladder carcinoma and predicted poor survival. The overexpression of lncRNA-HGBC promoted the proliferation and invasion of gallbladder carcinoma both *in vitro* and *in vivo*. LncRNA-HGBC functioned as a ceRNA to competitively bind the miR-502-3p that inhibited the target gene *SET* (17). The lncRNA JPX was highly expressed in lung cancer metastatic tissues and was positively correlated with tumor size and tumor stage. JPX promoted lung cancer cell growth and invasion *via* the JPX/miR-33a-5p/Twist1 axis and by activating Wnt/β-catenin signaling (34).

In this study, we confirmed that the lncRNA LGALS8-AS1 served as an oncogenic lncRNA that strengthens metastasis in breast cancer. By searching the RNA sequence data from TCGA dataset, we identified that LGALS8-AS1 was dysregulated in breast cancer tissues. In addition, we verified LGALS8-AS1 to be highly expressed in both breast cancer cell lines and tissues by RT-qPCR. The Transwell and wound healing assays and the lung metastasis models further proved that LGALS8-AS1 was related to breast tumor metastasis. Subsequently, we used the TargetScan online database and found that LGALS8-AS1 could function as a ceRNA for miR-125b-5p, consequently counteracting the effects of miR-125b-5p on the migration and invasion of breast cancer *in vitro*. miR-125b-5p has been reported as a tumor-suppressive miRNA in some tumor types. Morelli et al. proved miR-125b-5p to have tumor-suppressor activity in multiple myeloma (MM) *via* directly downregulating *IRF4* and BLIMP-1 (35). Resveratrol regulates the apoptosis and cell cycle in breast cancer *via* modulating miR-125b-5p (36).

We next searched for the downstream targets of miR-125b-5p and found that the expression of SOX12 was negatively related to miR-125b-5p. Moreover, it was positively related to lncRNA-LGALS8-AS1. SOX12 played an important role in tumor proliferation and metastasis and was increased in different types of cancers (from TCGA database, data not shown). SOX12 knockdown could inhibit the proliferation of leukemia cells *in vitro* and suppressed the leukemia initiation in a mouse model (37). It was reported that SOX12 played a crucial role in the maintenance of hepatocellular carcinoma metastasis (38). Increased expression of SOX12 was related to gastric cancer cell migration, invasion, and metastasis *via* transcriptionally targeting matrix metallopeptidase 7 (MMP7) and insulin-like growth factor 1 (IGF1) (27). In addition, the IGF1 feedback loop promoted SOX12 expression *via* the PI3K/AKT/CREB pathway.

We analyzed the LGALS8-AS1 promoter and found that the transcription factor SOX12 promoted LGALS8-AS1 transcription. Cells transfected with a luciferase reporter construct containing a SOX12 binding enrichment motif were transfected with the SOX12 overexpression plasmid and showed significantly lower luciferase activity than those transfected with the control vector (**Figure 7D**). This finding indicated that LGALS8-AS1 could be feedback loop transcriptionally activated by SOX12. We also performed GSEA based on the dataset from TCGA and concluded that LGALS8-AS1 expression was related to the PI3K/AKT signaling pathway, which was further verified by Western blot. These results were consistent with previous studies showing that the activation of the PI3K/AKT/mTOR pathway was usually associated with tumor migration and invasion (39, 40).

In conclusion, our results revealed a novel metastatic molecular mechanism in breast cancer. The LGALS8-AS1/miR-125b-5p/SOX2 reciprocal regulatory loop dyscrasia promoted the migration and invasion of breast cancer cells. This signaling axis could be applicable to the design of novel therapeutic strategies against this malignancy.

## Data Availability Statement

The original contributions presented in the study are included in the article/**Supplementary Material**. Further inquiries can be directed to the corresponding authors.

## Ethics Statement

The animal study was reviewed and approved by Southern Medical University Experimental Animal Ethics Committee (STAMP).

## Author Contributions

DZ and YL conceptualized the study. RY, TL, and JB helped with the methodology. XK and SN contributed to the validation. JB and DZ performed formal analysis. TL curated the data. DZ prepared the original draft. DZ and TL reviewed and edited the manuscript. RY and XK helped with the visualization. YS polished the manuscript. YL supervised the study. YL administered the project. YL and SN helped with funding acquisition.

## Funding

This work is supported by the National Natural Foundation of China (81902675) and the Natural Science Foundation of Guangdong Province (2021A1515010333).

## Conflict of Interest

The authors declare that the research was conducted in the absence of any commercial or financial relationships that could be construed as a potential conflict of interest.

The reviewer HT declared a shared affiliation with the authors to the handling editor at the time of the review.

## Publisher’s Note

All claims expressed in this article are solely those of the authors and do not necessarily represent those of their affiliated organizations, or those of the publisher, the editors and the reviewers. Any product that may be evaluated in this article, or claim that may be made by its manufacturer, is not guaranteed or endorsed by the publisher.

## Supplementary Material

Supplementary Figure 1 LGALS8-AS1 promotes the metastasis of breast cancer cells. **(A)** Transfection efficiency of LGALS8-AS1 shRNA and overexpression plasmid detected by RT-qPCR in MDA-MB-231 and MCF-7 cells. **(B)** The effect of LGALS8-AS1 overexpression on cell migration and invasion of MDA-MB-231 and MCF-7 cells determined by transwell assays. **(C)** The effect of LGALS8-AS1 overexpression on cell migration and invasion of MDA-MB-231 and MCF-7 cells determined by the scratch assay. ****p* < 0.001.

Supplementary Figure 2 LGALS8-AS1 promoted SOX12 mediated metastasis through decoying hsa-miR-125b-5p. **(A)** The effect of LGALS8-AS1 inhibition or overexpression, together with miR-125b-5p mimics on expression of SOX12 was detected in MCF-7 cells by RT-qPCR. **(B-C)** The combine effect of LGALS8-AS1 inhibition and miR-125b-5p mimics or SOX12 overxpression on cell migration and invasion of MCF-7 cells determined by transwell and scratch assays. **p* < 0.05, ****p* < 0.001.

Supplementary Figure 3 Transfection efficiency of LGALS8-AS1 shRNA and LGALS8-AS1 overxpression plasmid. **(A)** Transfection efficiency of LGALS8-AS1 shRNA and LGALS8-AS1 overexpression plasmid for inhibition or overexpression detected by RT-qPCR assays in MDA-MB-231 and MCF-7 cells. ****p* < 0.001.

Supplementary Table 1 Primers for RT-qPCR.

Supplementary Table 2 Information of antibodies.
