# Supplementary material for: LncRNA LGALS8-AS1 Promotes Breast Cancer Metastasis Through miR-125b-5p/SOX12 Feedback Regulatory Network
Source: Front Oncol. 2021 Oct 22;11:711684. doi: 10.3389/fonc.2021.711684 (PMC8570098; doi:10.3389/fonc.2021.711684)
Supplement: Supplementary Figure 1 — LGALS8-AS1 promotes the metastasis of breast cancer cells. (A) Transfection efficiency of LGALS8-AS1 shRNA and overexpression plasmid detected by RT-qPCR in MDA-MB-231 and MCF-7 cells. (B) The effect of LGALS8-AS1 overexpression on cell migration and invasion of MDA-MB-231 and MCF-7 cells determined by transwell assays. (C) The effect of LGALS8-AS1 overexpression on cell migration and invasion of MDA-MB-231 and MCF-7 cells determined by the scratch assay. ***p < 0.001. [file DataSheet_1.docx]

Supplementary Material

# Supplementary Figures and Tables

## Supplementary Figures


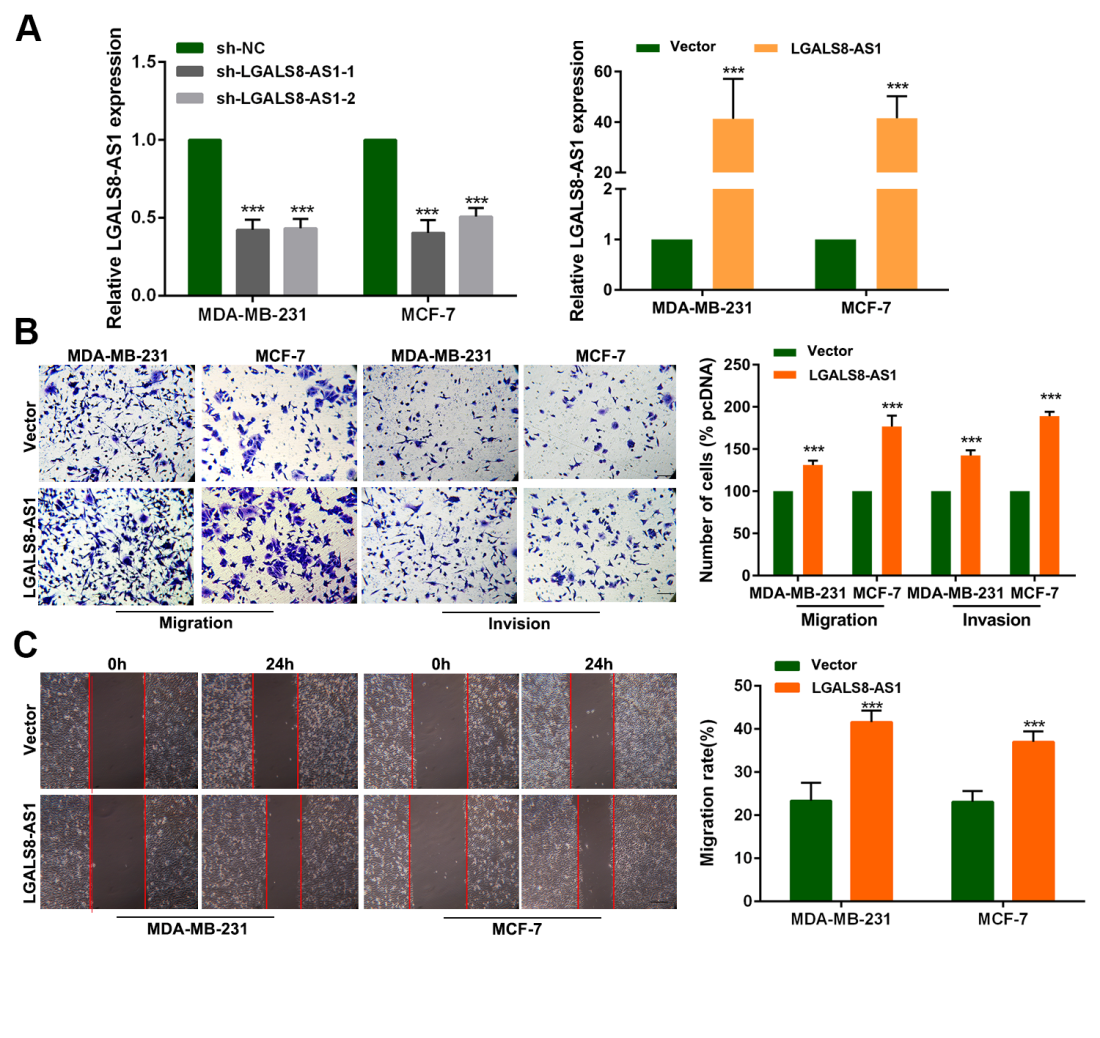


**Supplementary Figure 1. LGALS8-AS1 promotes the** **metastasis of breast cancer cells.** **(A)** Transfection efficiency of LGALS8-AS1 shRNA and LGALS8-AS1 overxpression plasmid for inhibition or overexpression detected by RT-qPCR assays in MDA-MB-231 and MCF-7 cells. **(B)** The effect of LGALS8-AS1 overexpression on cell migration and invasion of MDA-MB-231 and MCF-7 cells determined by transwell assays. **(C)** The effect of LGALS8-AS1 overxpression on cell migration and invasion of MDA-MB-231 and MCF-7 cells determined by the scratch assay. **p*<0.05, ***p*<0.01, ****p*<0.001.


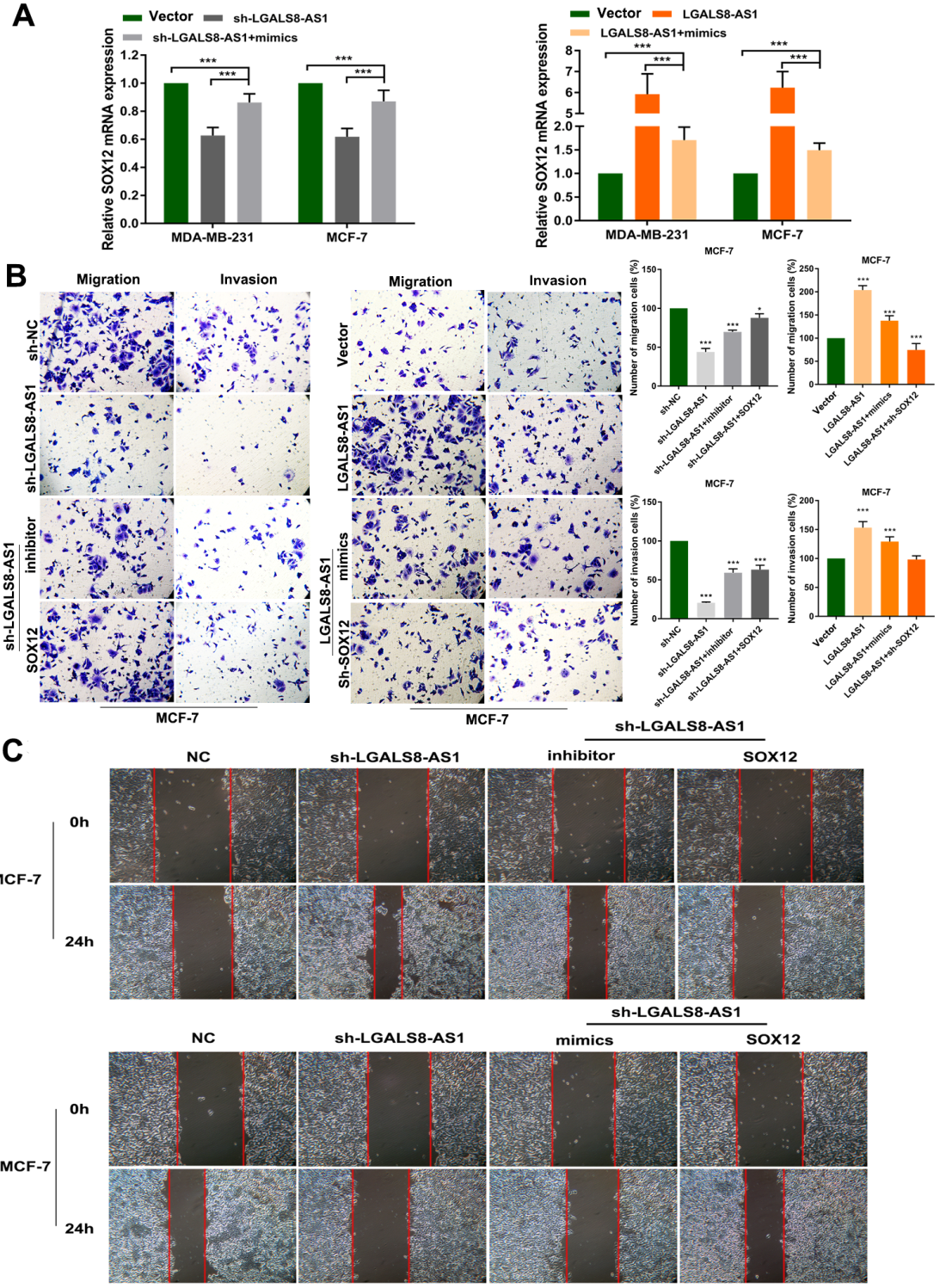


**Supplementary Figure 2. LGALS8-AS1 promoted SOX12 mediated metastasis through decoying hsa-miR-125b-5p.** **(A)** The effect of LGALS8-AS1 inhibition or overxpression,together with miR-125b-5p mimics on expression of SOX12 was detected in MCF-7 cells by RT-qPCR. **(B-C)** The combine effect of LGALS8-AS1 inhibition and miR-125b-5p mimics or SOX12 overxpression on cell migration and invasion of MCF-7 cells determined by transwell and scratch assays. **p* < 0.05, ** *p*<0.01, ****p*<0.001.


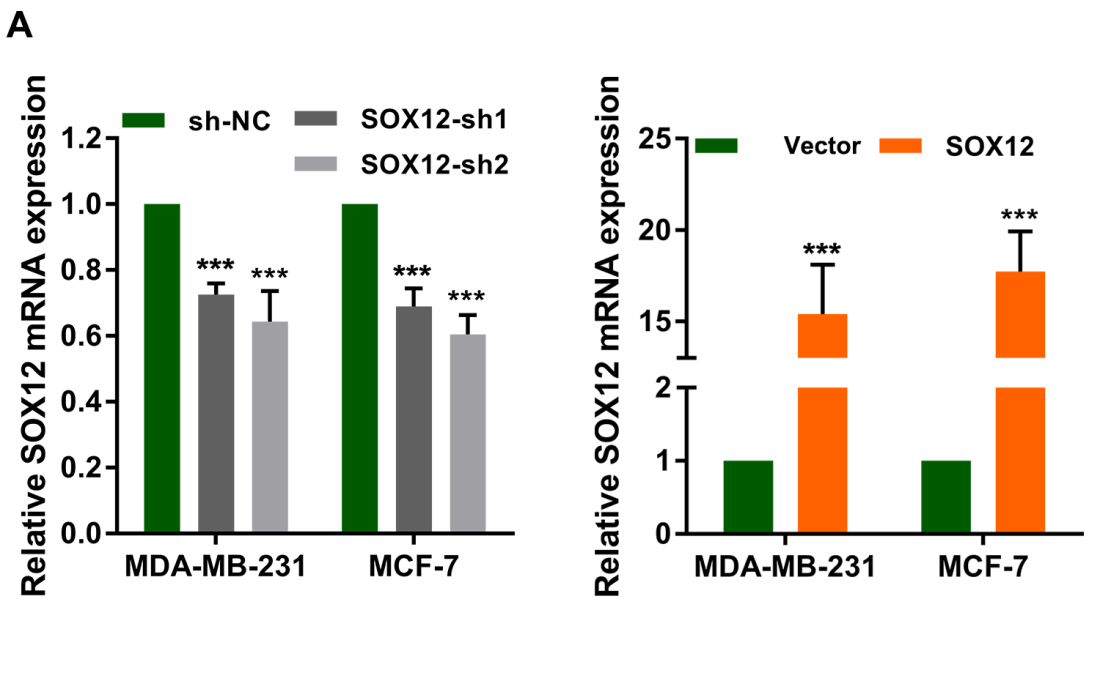


**Supplementary Figure 3. Transfection efficiency of LGALS8-AS1 shRNA and LGALS8-AS1 overxpression plasmid.** (**A)** Transfection efficiency of LGALS8-AS1 shRNA and LGALS8-AS1 overxpression plasmid for inhibition or overexpression detected by RT-qPCR assays in MDA-MB-231 and MCF-7 cells. **p*<0.05, ** *p*<0.01, ****p*<0.001.

## Supplementary Tables

**Supplementary table 1 Primers for RT-qPCR**

| Genes | Primers |
| --- | --- |
| SOX12 | 5ʹ- GACATGCACAACGCCGAGATCT-3ʹ (forward) |
|  | 5ʹ- GTAATCCGCCATGTGCTTGAGC-3ʹ (reverse) |
| GADPH | 5ʹ- GTCTCCTCTGACTTCAACAGCG-3ʹ (forward) |
|  | 5ʹ- ACCACCCTGTTGCTGTAGCCAA-3ʹ (reverse) |
| U6 | 5ʹ- CTCGCTTCGGCAGCACATATACT-3ʹ (forward) |
|  | 5ʹ-ACGCTTCACGAATTTGCGTGTC-3ʹ (reverse) |
| miR-125b-5p | 5′-TCCCTGAGACCCTAACTTGTGA-3′ (forward) |
|  | 5′- AGTCTCAGGGTCCGAGGTATTC-3′ (reverse) |
| LGALS8-AS1 | 5′-ACATCCGAATGCCATCCTCC-3′ (forward) |
|  | 5′-AGGACTGACTCCTGTCGCTT-3′ (reverse) |

**Supplementary table 2 Information of antibodies**

| Antibody | Product number | Dilution ratio |
| --- | --- | --- |
| anti-SOX12 | ab54371, Abcam, USA | 1:1000 |
| anti-GAPDH | ab8245, Abcam, USA | 1:1000 |
| anti-AKT | 4691S, CST, USA | 1:1000 |
| anti-p-AKT | 4060S, CST, USA | 1:1000 |
| anti-N-cadherin | 13116T CST, USA | 1:500 |
| anti-E-cadherin | 14472S, CST, USA | 1:500 |
| anti-Vimentin | 5741T CST, USA | 1:500 |
| anti-SNAIL1 | 3879S CST, USA | 1:500 |
| anti-p-P85 | ab138364, Abcam, USA | 1:1000 |
